# Serum RANKL levels associate with anti- citrullinated protein antibodies in early untreated rheumatoid arthritis and are modulated following methotrexate

**DOI:** 10.1186/s13075-015-0760-9

**Published:** 2015-09-04

**Authors:** Aase Haj Hensvold, Vijay Joshua, Wanying Li, Michaela Larkin, Ferhan Qureshi, Lena Israelsson, Leonid Padyukov, Karin Lundberg, Nadine Defranoux, Saedis Saevarsdottir, Anca Irinel Catrina

**Affiliations:** Rheumatology Unit, Department of Medicine, Karolinska Institutet, Karolinska University Hospital, L8:04 CMM, 171 76 Stockholm, Sweden; Crescendo Bioscience, 341 Oyster Point Boulevard, South San Francisco, CA 94080 USA; Institute of Environmental Medicine, Karolinska Institutet, Solnavägen 1, 171 77 Stockholm, Sweden

## Abstract

**Introduction:**

Receptor activator of nuclear factor kappa B ligand (RANKL) is a key regulator of bone metabolism. Anti-citrullinated protein antibodies (ACPA) have been suggested to cause bone destruction by osteoclast activation. We investigated the relationship between RANKL and ACPA in patients with early untreated rheumatoid arthritis (RA).

**Methods:**

Patients with newly diagnosed untreated RA (n = 183) were analyzed at baseline and 3 months after initiating methotrexate (MTX) treatment. Serum RANKL (total RANKL), ACPA (anti-CCP2) and ACPA specificities (anti-citrullinated (cit)-vimentin, anti-cit-enolase and anti-cit-fibrinogen) were determined by enzyme-linked immunosorbent assay (ELISA). Synovial RANKL expression was evaluated by immunohistochemistry in a small group of patients (n = 15). The relationship between anti-cit-vim antibodies and bone destruction was further validated in 1116 RA patients included in the EIRA cohort. Pearson’s chi-square test, Wilcoxon rank sum test, Wilcoxon signed rank test and linear regression models were used.

**Results:**

Serum RANKL concentration was significantly higher (*p* <0.05) in ACPA-positive (median: 689 pmol/L, IQR 342–1253) compared with ACPA-negative (median: 159 pmol/L, IQR 96–243) patients and this difference was also seen for synovial RANKL expression. Serum RANKL associated with ACPA (*p* <0.05) and bone erosions in rheumatoid factor (RF)-negative patients (n = 59). Among ACPA specificites, anti-cit-vimentin (amino acids 60–75) was associated with higher RANKL concentration and higher prevalence of bone erosion (*p* <0.05). Significant reductions in both serum RANKL and ACPA levels were observed after 3 months of MTX treatment (*p* <0.05).

**Conclusions:**

RANKL was elevated in ACPA-positive and in anti-cit-vimentin-positive patients with early untreated RA and associated with bone erosions. These findings give further support for an early direct pathogenic link between ACPA and bone destruction in RA.

## Introduction

Osteoimmunology is a conceptual and molecular understanding of how the immune system influences the bone metabolism in diseases such as rheumatoid arthritis (RA) [1, 2]. RA is a chronic inflammatory disease affecting the synovial membrane of the joints and bone [3, 4]. Approximately half of the patients, with symptom duration of less than 1 year, present with radiographic bone damage in small joints at diagnosis [5, 6]. Presence of systemic autoimmunity with rheumatoid factor (RF) and/or anti-citrullinated protein antibodies (ACPA) in RA is associated with an increased risk of bone damage [7–10]. Recently, a new cellular mechanism has been suggested by which ACPA specifically increase bone destruction in RA. According to this, ACPA binding to the surface of osteoclast precursors increases their number, possibly by stimulation of tumor necrosis factor alpha (TNF-α) production [11]. In addition to ACPA, markers of inflammation and of high disease activity (e.g., C-reactive protein (CRP) and disease activity score (DAS) 28) have also been shown to be associated with increased bone damage in patients with RA [8, 10]. Efficient treatment with disease-modifying antirheumatic drugs (DMARD), including methotrexate (MTX), results in reduced disease activity and less bone destruction [12], while the effect on ACPA is still not completely elucidated [13, 14].

Receptor activator of nuclear factor kappa B ligand (RANKL) is in the concept of osteoimmunology; a key molecule in the regulation of bone metabolism and the linkage between immune and skeletal systems [15, 16]. RANKL is affected by proinflammatory cytokines such as TNF-α, interleukin (IL)-1 and IL-6 [4] and has been suggested to be a marker of bone damage in RA [17–20]. However, the linkage between immune system and the influence of ACPA immunity on RANKL in early RA is largely unexplored. RANKL is expressed in synovial tissue [18, 21, 22] and serum [6, 23, 24] but no studies on RANKL’s relationship to ACPA status have been previously conducted in untreated RA.

In this study, we aimed to determine to what extent RANKL levels associate with presence of ACPA, bone erosions and MTX treatment in a cohort of patients with early untreated RA.

In summary, we can report that RANKL was elevated in ACPA-positive and in anti-cit-vimentin-positive patients and associated with bone erosions in patients with early untreated RA.

## Methods

### Patients

The study was performed in a cohort of 183 patients with early untreated RA with symptom onset <1 year prior to diagnosis, recruited at the Rheumatology Clinic at Karolinska University Hospital, Stockholm (during years 1996–2006) and part of the Epidemiological Investigation of Rheumatoid Arthritis (EIRA) study cohort [25]. Clinical data and data on rheumatoid factor (RF) positivity were obtained from the Swedish Rheumatology quality registers. All patients in this study started on MTX, with or without concomitant nonsteroidal anti-inflammatory drugs (NSAID) and/or prednisolone, to a final dose of 10–20 mg/week following the local guidelines. Regarding antiporotic treatment: 10 out of 181 patients (13 %, 2 with missing data) were on calcium and/or vitamin D supplements and 16 out of 181 (9 %, 2 with missing data) on hormone replacement therapy, while none was treated with either bisphosphonates or denosumab at inclusion. An additional number of 10 out of 181 patients (5 %) and 1 out of 181 patients (1 %) were prescribed calcium and/or vitamin D supplement, respectively, and/or bisphosphonates at inclusion.

Serum samples and DAS28 based on the erythrocyte sedimentation rate (ESR) were obtained at baseline and at clinical follow-up, which occurred after a median of 14 weeks (interquartile range 25−75 % (IQR) 13−15). Data on the presence of HLA-DRB1 shared epitope (SE) gene allele, protein tyrosine phosphatase gene allele (PTPN22 rs2476601) and body mass index (BMI) at inclusion were also available [26–28].

Review of the medical records provided information on the presence or absence of at least one bone erosion at baseline, based on standard radiographs of hands and feet according to clinical routine at our unit. Seven patients were excluded from posttreatment analyses due to delayed MTX treatment initiation (>7 weeks after baseline) or secession of treatment; these patients were, however, included in pretreatment (baseline) analyses.

Additionally, 1116 patients from the EIRA study cohort with available study data on standard radiograph changes in hands (at least one change according to 1987 RA criteria [29]) and serum ACPA status [30], were selected as a validation cohort. Patients in this validation cohort had a median age of 54 years; 69 % were females, 62 % (n = 687) ACPA-positive and 36 % (n = 405) anti-citrullinated vimentin (cit-vim)-positive. The Ethics Review Board at Karolinska Institutet approved the study and an informed consent was obtained from participants.

### Immunoassays

ACPA were detected by anti-cyclic citrullinated peptide version 2 (anti-CCP2) test (CCPlus Immunoscan, Malmö, Sweden) using the manufacturer’s protocol. ACPA levels above the highest standard sample (>3200 AU/ml) were reassessed after appropriate dilution. Antibodies (anti)-cit-enolase peptide 1 (CEP-1, amino acids 5–21), anti-cit-vim peptide (amino acids 60–75), and anti-cit-fibrinogen (fib) peptide (amino acids 563–583) were measured in sera by previously established enzyme-linked immunosorbent assay (ELISA). Native forms of the same peptides were used as controls [31]. Serum concentrations of the total (bound and unbound) soluble RANKL were measured by ELISA (Biovendor, Brno, Czech Republic) in accordance with the manufacturer’s instructions. Serum concentrations of CRP, IL-6, and tumor necrosis factor receptor type 1 (TNF-RI) were measured by multiplex sandwich immunoassay (Sector Imager 6000, Meso Scale Discovery, Gaithersburg, MD, USA) [32]. Paired samples (before and after treatment) were run at the same assay and handled the same way.

### Synovial biopsies and immunohistochemistry

Synovial biopsies obtained during arthroscopy were available from 15 patients with early untreated RA (including 7 of the 183 patients that donated blood). Patients had a median age of 56 years (range 33–78); 10 out of 15 were females; 7 out of 15 were ACPA-positive and 2 out of 15 had bone erosion at baseline. Synovial expression of RANKL was detected by immunohistochemistry staining, using a monoclonal anti-human RANKL detection antibody at a final concentration of 5 μg/ml (12A668, Abcam, Cambridge, UK) as previously described [22, 33]. The RANKL expression level was evaluated using computer-assisted image analysis and expressed as the percentage of the total tissue area that stained positive.

### Statistical analysis

Pearson’s chi-square test, Wilcoxon rank sum test and Wilcoxon signed rank test were used for independent and paired comparisons as appropriate. To investigate the relationship between RANKL and ACPA independent of RF, the analysis of RANKL was performed both in the overall group (n = 183) and also in the subset of RF-negative patients (n = 59).

In order to investigate the association between RANKL concentration (logarithm-transformed concentration) and ACPA status, we also tested other possible predictors for RANKL: age, sex, smoking habits (history of ever or never smoking), BMI, DAS28-ESR, ESR, CRP, IL-6 serum levels, TNF-RI serum levels, health assessment questionnaire (HAQ) values, concurrent prednisolone usage, concurrent usage of antiporotic treatment, presence of HLA-DRB1 SE, presence of PTPN22 risk allele, one by one in univariate linear regression models. A multiple regression model for the association between RANKL and ACPA was then obtained by including the significant predictors from the univariate analyses. Statistical analyses were conducted using SAS 9.3 (SAS Institute Inc., Cary, NC, USA). Two tailed *p* values <0.05 were considered statistically significant. No adjustments for multiple testing were performed in the explorative part of the study, but Bonferroni correction was applied for comparisons in the validation cohort.

## Results

### Increased RANKL levels in ACPA-positive untreated early RA

Of the 183 patients, 125 (68%) were ACPA-positive with a median concentration of 752 AU/ml (IQR 278–2174). Among these ACPA-positive patients 58% were also positive for anti-cit-enolase, 52% for anti-cit-vim, and 31% for anti-cit-fib (see Table 1).

**Table 1:** Characteristics at baseline of the rheumatoid arthritis cohort

	ACPA-positive	ACPA-negative	ACPA-positive	ACPA-negative
			RF-negative	RF-negative
Numbers	125	58	14	45
Age, years	48 (40–56)	58 (52–64)	43 (29–50)	59 (55–65)
Female sex	97 (78 %)	35 (60 %)	11 (79%)	27 (60 %)
Ever smoking	71 (66 %)	36 (67 %)	7 (64 %)	28 (67 %)
HLA-DRB1 SE	109 (89 %)	34 (60 %)	13 (100 %)	25 (57 %)
PTPN22 risk allele	40 (33 %)	10 (18 %)	5 (38 %)	6 (14 %)
DAS28-ESR	5.6 (5.0–6.2)	5.8 (5.1–6.3)	6.0 (5.2–6.2)	5.9 (5.1–6.3)
ESR	28 (16–40)	23 (12–39)	28 (25–35)	21 (12–35)
HAQ	1.1 (0.8–1.6)	1.3 (0.9–1.5)	1.0 (0.8–1.5)	1.3 (0.9–1.5)
Prednisolone	16 (13 %)	10 (18 %)	1 (7 %)	7 (16 %)
Calcium and/or vitamin D supplement	7 (6 %)	3 (5 %)	1 (7 %)	1 (2 %)
Hormone replacement therapy	9 (7 %)	7 (12 %)	0 (0 %)	5 (11 %)
RF-positive	111 (89 %)	13 (22 %)		
Anti-cit-enolase (amino acids 5–21)+	73 (58 %)	0 (0 %)	6 (43 %)	0 (0 %)
Anti-cit-vimentin (amino acids 60–75)+	65 (52 %)	0 (0 %)	7 (50 %)	0 (0 %)
Anti-cit-fibrinogen (amino acids 563–583)+	39 (31 %)	1 (2 %)	2 (14 %)	1 (2 %)

Values are given as number (%) or median (25–75% interquartile range: (IQR))

Number of missing observations for smoking habits, HLA-DRB1 SE, PTPN22, use of calcium and/or vitamin D, use of hormone replacement therapy, use of prednisolone and HAQ, were 22, 3, 5, 2, 2, 5 and 4 respectively

*ACPA* anti-citrullinated protein antibodies, *RF* rheumatoid factor, *DAS28* disease activity score 28, *ESR* erythrocyte sedimentation rate (mmHg/hour), *HAQ* health assessment questionnaire (range: 0–3), *cit* citrullinated, *+* positive

**Fig. 1:**
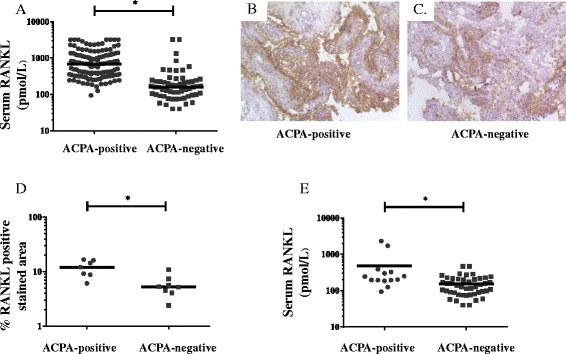
Serum and synovial RANKL is increased in ACPA-positive as compared to ACPA-negative rheumatoid arthritis (RA). Graphs illustrate the results of ELISA measurement of total serum RANKL concentrations in RA (**a**) and in RF-negative RA (**e)** grouped by ACPA status. Immunohistochemistry staining shows expression of synovial RANKL in one ACPA-positive (**b**) and one ACPA-negative RA patient (**c**) and the graph illustrate the results of image analysis in 15 patients (**d**). Horizontal lines represent median values, ^*^ *p* <0.05. *ACPA* anti-citrullinated protein antibodies, *ELISA* enzyme-linked immunosorbent assay, *RANKL* receptor activator of nuclear factor kappa B ligand, *RF* rheumatoid factor

Serum concentration of RANKL (Fig. 1a) was significantly higher in ACPA-positive (689 pmol/L, IQR 342–1253) than in ACPA-negative patients (159 pmol/L, IQR 96–243, *p* <0.05). Similarly, median synovial expression of RANKL (Fig. 1b-d) was significantly higher in ACPA-positive than in ACPA-negative patients.

To further investigate the relationship between RANKL and ACPA independent of RF, we performed a separate analysis of RF-negative patients. Similar to the whole cohort, the median RANKL serum concentration remained significantly higher in RF-negative ACPA-positive (222 pmol/l, IRQ 194–327, n = 14) as compared to RF-negative ACPA-negative patients (128 pmol/l, IRQ 88–207, n = 45, *p* <0.05) (Fig. 1e). Significantly higher RANKL concentrations were also observed for those who were anti-cit-enolase positive (272 pmol/l, IQR 194–327, n = 6) or anti-cit-vim positive (244 pmol/l, IQR 194–388, n = 7) compared with those who were anti-cit-enolase negative (146 pmol/l, IQR 91–216, n = 53, *p* <0.05) or anti-cit-vim negative (151pmol/l, IQR 90–217, n = 52, *p* <0.05), respectively.

Using linear univariate regression models we identified significant association between serum RANKL and ACPA, age, DAS28-ESR and BMI, while all other tested variables (sex, smoking habits, ESR, CRP, IL-6 serum levels, TNF-RI serum levels, HAQ values, use of prednisolone or antiporotic treatment, presence of HLA-DRB1 SE and PTPN22 risk allele) were not significant predictors for RANKL. Only ACPA and BMI remained significant in the multivariate model. A mean of 232 pmol/l (95 % CI: 155–346) RANKL in ACPA positive and 140 pmol/l (95 % CI: 114–171) RANKL in ACPA negative was estimated after adjustments of age, DAS28-ESR and BMI (Table 2).

**Table 2:** Linear regression models showed unadjusted and adjusted association between RANKL concentration and ACPA

	Least square mean of RANKL pmol/L		Coefficient	*p* value	R^2^
	(95% CI)				(adj R^2^)
	ACPA-	ACPA-			
	positive	negative			
Model A. (n = 59)	290	130			0.21
	(201–417)	(106–160)			(0.19)
ACPA			0.35	<0.001	
(positive vs. negative)					
*Intercept*			2.12		
Model B. (n = 59)	232	140			0.36
	(155–346)	(114–171)			(0.31)
ACPA			0.22	0.04	
(positive vs. negative)					
Age			-0.005	0.17	
(per 1-year increase)					
DAS28-ESR			0.07	0.06	
BMI			-0.02	0.04	
*Intercept*			2.53		

R^2^: proportion of variance explained by the variables in the statistical model

adj R^2^: adjusted R^2^, similar to R^2^ but takes into account the number of variables in the model

*RANKL* receptor activator of nuclear factor kappa B ligand, *ACPA* anti-citrullinated protein antibodies, *DAS28* disease activity score 28, *ESR* erythrocyte sedimentation rate, *BMI* body mass index

### Serum RANKL and ACPA associate with bone erosion in untreated early RA

**Fig. 2:**
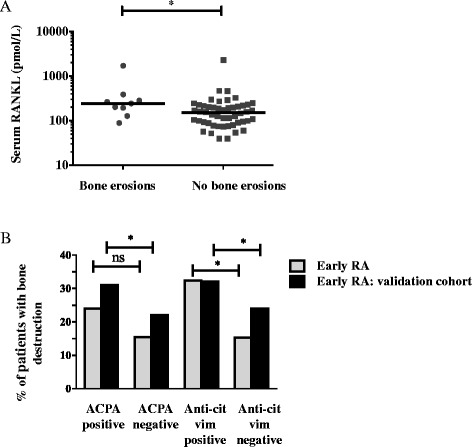
Serum RANKL and ACPA associate with bone destruction. Graphs show the results of ELISA measurement of total serum RANKL concentrations in RF-negative RA patients grouped by bone erosion status (**a**). ACPA-positive and anti-cit-vim-positive patients observed higher prevalence of bone destructions than ACPA-negative or anti-cit-vim-negative patients in both early RA cohorts (**b**). Horizontal lines represent median values, ^*^ *p* <0.05; ns: *p* >0.05. VC denotes validation cohort. *ACPA* anti-citrullinated protein antibodies, *cit* citrullinated, *ELISA* enzyme-linked immunosorbent assay, *RA* rheumatoid arthritis, *RANKL* receptor activator of nuclear factor kappa B ligand, *RF* rheumatoid factor, *vim* vimentin

RANKL serum concentrations were significantly elevated (*p* <0.05) in patients with evidence of bone erosions at baseline (median 243 pmol/l, IQR 194–284, n = 9) compared with those without bone erosions (151 pmol/l, IQR 91–216, n = 50) (Fig. 2a). Baseline bone erosions were numerically more prevalent in ACPA-positive than ACPA-negative patients (24 vs. 16 %, *p* >0.05) and significantly more prevalent in anti-cit-vim (amino acids 60–75) -positive than anti-cit-vim-negative patients (32 vs. 15 %, *p* <0.05) (Fig. 2b), while no such differences were observed for anti-cit-enolase (amino acids 5–21) or anti-cit-fib (amino acids 563–583) antibodies. Interestingly, this association appear to be independent of ACPA levels as far as anti-cit vim (amino acids 60–75) -positive patients had a median ACPA titer of 533 AU/ml (IQR 190–1742), while anti-cit-fib (amino acids 563–583) -positive patients had a significant higher median ACPA titer of 2286 AU/ml (IQR 1580–5885).

The association between anti-cit-vim (amino acids 60–75) antibodies and bone destruction finding was confirmed in the validation cohort (n = 1116) in which we found, that both ACPA-positive and anti-cit-vim (amino acids 60–75) -positive patients had a higher frequency of destructions in comparison to ACPA-negative (31 vs. 22 %, Bonferroni-corrected *p* value <0.05) and anti-cit-vim-negative patients (32 vs. 24 %, Bonferroni-corrected *p* value <0.05), respectively (Fig. 2b).

### Decreased serum RANKL levels after MTX treatment

Serum RANKL concentrations decreased significantly following MTX treatment from baseline to 3 months (a median decrease of 58 pmol/l, IQR 8–276, *p* <0.05) and the changes were similar in the subset of RF-negative patients. Serum RANKL levels decreased in a large majority of the patients (83 %, Table 3). Significant changes were observed in both RF-negative ACPA-positive patients (a median decrease of 37 pmol/l, IQR 5–68, *p* <0.05) and RF-negative ACPA-negative patients (a median decrease of 14 pmol/l, IQR 3–38, *p* <0.05). Interestingly, RANKL concentration after 3 months of MTX treatment remained higher in RF-negative ACPA-positive patients (202 pmol/l, IQR 133–322) than in RF-negative ACPA-negative patients (111 pmol/l, IQR 75–180, *p* <0.05).

**Table 3:** Number of patients with decreased or increased levels of RANKL, IL-6, TNF-RI, ACPA and ACPA fine specificities at follow-up

	Number of patients with decreased levels	Number of patients with unchanged or increased levels	Total number of patients
RANKL	145 (83 %)	29 (17 %)	174
IL-6	120 (68 %)	56 (32 %)	176
TNF-RI	124 (70 %)	52 (30 %)	176
ACPA	107 (89 %)	13 (11 %)	120
Anti-cit-enolase (amino acids 5–21)	58 (82 %)	13 (18 %)	71
Anti-cit-vimentin (amino acids 60–75)	59 (88 %)	8 (12 %)	67
Anti-cit-fibrinogen (amino acids 563–583)	35 (83 %)	7 (17 %)	42

Values are given as number (%). We report change in antibody concentrations for patients being positive at baseline or at 3 month for the corresponding antibody

*RANKL* receptor activator of nuclear factor kappa B ligand, *IL* interleukin, *TNF-RI* tumor necrosis factor receptor type I, *ACPA* anti-citrullinated protein antibodies, *cit* citrullinated

### Decreased serum ACPA concentrations after MTX treatment

**Fig. 3:**
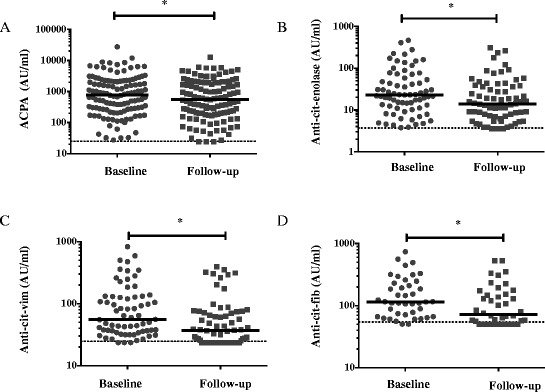
Serum concentration of ACPA and ACPA specificities. Graphs show the results of ELISA measurements of the antibody concentrations for patients being positive at baseline or at 3 months for the corresponding antibody: ACPA n = 120 (**a**), anti-cit-enolase (amino acids 5–21) n = 71 (**b**), anti-citrullinated (cit)-vimentin (vim) (amino acids 60–75) n = 67 (**c**), anti-cit-fibrinogen (fib) (amino acids 563–583) antibodies n = 42 (**d**). Horizontal lines represent median values, ^*^ *p* <0.05. Dotted lines delineate ELISA cutoff values for each antibody. *ACPA* anti-citrullinated protein antibodies, *ELISA* enzyme-linked immunosorbent assay

ACPA concentration decreased from 780 AU/ml (IRQ 273–2197) at baseline to 556 (IRQ 197–1920) at 3 months (*p* <0.05), for the patients that were ACPA-positive at baseline and/or at 3 months (n = 120) (Fig. 3a). ACPA levels decreased in a large majority of the patients (89 %, Table 3).

Median concentrations were also observed to decrease from baseline to 3 months for anti-cit-enolase (amino acids 5–21) (from 23 AU/ml, IQR 11–58, to 14A U/ml, IQR 7–38, *p* <0.05), anti-cit-vim (amino acids 60–75) (from 56 AU/ml, IQR 36–130, to 37 AU/ml, IQR 25–74, *p* <0.05) and anti-cit-fib (amino acids 563–583) (from 115 AU/ml, IQR 66–230, to 72 AU/ml, IQR 52–153, *p* <0.05), for patients positive either at baseline or at 3 months (Fig. 3b-d). Significant decreases were also observed when the analysis was performed on the overall population (n = 176).

In overall population, antibody seroconversion from positive at baseline to negative at 3 months was observed for 3 of the 120 (2.5 %) ACPA-positive, 4 of the 71 (5.6 %) anti-cit-enolase (amino acids 5–21) -positive, 21 of the 64 (33 %) anti-cit-vim (amino acids 60–75) -positive and 12 of the 40 (30 %) anti-cit-fib (amino acids 563–583) -positive patients. Conversion from seronegative at baseline to seropositive at 3 months was observed infrequently: anti-cit-vim (amino acids 60–75) (3 out of 112) (2.7 %); anti-cit-fib (amino acids 563–583) (2 out of 136) (1.5 %). No patients who were ACPA or anti-cit-enolase (amino acids 5–21) seronegative at baseline converted to seropositive.

## Discussion

Bone destruction, essentially dependent on the effect of RANKL on osteoclast precursors, associates with the presence of ACPA in RA. ACPA directly affects osteoclast precursors, but the relationship between ACPA and RANKL has not yet been investigated. In this study, we report that serum and synovial RANKL levels, used as surrogate makers of local and systemic bone destruction, are higher in ACPA-positive than in ACPA-negative newly diagnosed untreated RA patients. Both ACPA and RANKL levels decrease following treatment with methotrexate (MTX),.

We show that RANKL serum levels are higher in ACPA-positive as compared to ACPA-negative patients with early untreated RA, in contrast to one previous study were they found difference in a degradation product of type I collagen (CTX-I) but not RANKL [34]. Detection of only free RANKL (in contrast to detection of both free and bound RANKL in our study) and/or inclusion of patients with treated established RA (in contrast to untreated early RA in our study) might explain this discrepancy. The results presented here are in line with a previous study showing higher levels of bone resorption markers (such as CTX-I, tartrate-resistant acid phosphatase 5b and cathespin K) in the serum of ACPA-positive as compared to ACPA-negative newly diagnosed untreated RA patients [11]. Notably, the differences in RANKL serum levels depending on ACPA status in our study are still detectable when the analysis was performed in the subgroup of RF-negative patients, eliminating the bias of RF interference. We detected relatively low differences in RANKL levels between erosive and nonerosive disease (in median a 40 % lower level in erosive as compared to nonerosive disease) and following therapy (in median a 20 % decrease following 3 months of treatment). However, differences of similar magnitude have previously been shown to be of biological relevance for RA-associated bone destruction [6]. Equally important the decrease in RANKL levels after 3 months therapy with MYX was observed in a majority of the patients (>80%) and is in accordance with previous reports showing that MTX decrease RANKL expression in both synovial tissue and synovial-derived biopsies [22, 35, 36].

None of the measured inflammatory parameters (ESR, CRP, IL-6 or TNF-RI, data not shown) associated with ACPA and bone erosion in contrast to RANKL, which suggests an association between RANKL, ACPA and bone erosion that is at least partially uncoupled from inflammation. One previous investigation of serum pre-RA samples (n = 79) failed to detect differences in RANKL serum levels as compared to controls [37] despite more recent reports showing signs of bone destruction in ACPA-positive individuals already before disease onset [38]. One possible explanation is that local bone metabolism and RANKL changes are only mirrored in the peripheral blood once they reach a certain threshold at the time of onset of clinical joint symptoms.

ACPA have previously been associated with presence of radiological bone destruction in early RA patients [10, 39–41] though other studies reported conflicting results [7, 42–48]. Here, we confirmed, in one large early RA cohort (n = 1299 in total) the association of ACPA and presence of bone destruction. Interestingly we were not able to detect any clear association between levels of ACPA and bone destruction. In contrast, presence of anti-cit-vim (amino acids 60–75) antibodies associated with increased bone destruction and higher levels of serum RANKL, suggesting that these antibodies might contribute to the early bone changes observed in ACPA-positive individuals before disease onset [38].

Validation of our findings in other cohorts, testing for antibodies against more cit-peptides and investigation of the direct in vitro effect of different ACPAs specificities on osteoclastogenesis and bone metabolism are needed. We chose to define erosive disease as presence of at least one erosion on X-rays of hands and feet, allowing increased detection sensitivity in this cohort of patients already fulfilling the 1987 ACR classification criteria, in accordance with recently published recommendations from a EULAR task force [49].

However, a future more detailed analysis of bone changes (more sensitive techniques, quantification of the changes, separate analyses of cartilage and bone mass) not available in the current study might reveal more specific associations.

Lastly, serum RANKL, ACPA and ACPA specificities (anti-cit-enolase (amino acids 5–21), anti-cit-vim (amino acids 60–75), and anti-cit-fib (amino acids 563–583)) levels significantly decreased after 3 months of treatment with MTX and a relative high proportion of patients converted from seropositive to seronegative. These results are consistent with previous studies showing that MTX decreased synovial expression of RANKL in vivo, and cellular expression of RANKL in vitro [22, 35, 36]. Similar reduction in ACPA serum levels have been recently reported in early RA patients treated with either MTX in combination with sulfasalazine and hydroxychloroquine or MTX in combination with infliximab [14]. Taken together these results indicate that seroconversion from positive to negative ACPA might occur in patients following antirheumatic treatment. This should be taken into consideration when characterizing treated patients as antibody ‘seropositive’ or ‘seronegative’.

## Conclusions

In summary, serum RANKL in ACPA-positive early untreated RA associate with erosive disease and is modulated by MTX. Our findings give further support for an early direct pathogenic link between ACPA and bone destruction in RA.

## Abbreviations

ACPA: anti-citrullinated protein antibodies
anti-CCP2: anti-cyclic citrullinated peptide version 2
BMI: body mass index
cit: citrullinated
CRP: C-reactive protein
DAS28: disease activity score 28
DMARD: disease-modifying antirheumatic drug
EIRA: Epidemiological Investigation of Rheumatoid Arthritis
ELISA: enzyme-linked immunosorbent assay
ESR: erythrocyte sedimentation rate
fib: fibrinogen
HAQ: health assessment questionnaire
IL: interleukin
IQR: interquartile range
MTX: methotrexate
RA: rheumatoid arthritis
RANKL: receptor activator of nuclear factor kappa-b ligand
RF: rheumatoid factor
TNF-RI: tumor necrosis factor receptor type I
TNF-α: tumor necrosis factor alpha
vim: vimentin
